# Differences in Arithmetic Performance between Chinese and German Children Are Accompanied by Differences in Processing of Symbolic Numerical Magnitude

**DOI:** 10.3389/fpsyg.2016.01337

**Published:** 2016-08-31

**Authors:** Jan Lonnemann, Janosch Linkersdörfer, Marcus Hasselhorn, Sven Lindberg

**Affiliations:** ^1^Department of Education and Human Development, German Institute for International Educational Research (DIPF)Frankfurt am Main, Germany; ^2^Center for Individual Development and Adaptive Education of Children at RiskFrankfurt am Main, Germany; ^3^Department of Educational Psychology, Institute for Psychology, Goethe-UniversityFrankfurt am Main, Germany; ^4^Faculty of Arts and Humanities, Paderborn UniversityPaderborn, Germany

**Keywords:** magnitude comparison, symbolic number representation, arithmetic, cross-national comparison, elementary school

## Abstract

Symbolic numerical magnitude processing skills are assumed to be fundamental to arithmetic learning. It is, however, still an open question whether better arithmetic skills are reflected in symbolic numerical magnitude processing skills. To address this issue, Chinese and German third graders were compared regarding their performance in arithmetic tasks and in a symbolic numerical magnitude comparison task. Chinese children performed better in the arithmetic tasks and were faster in deciding which one of two Arabic numbers was numerically larger. The group difference in symbolic numerical magnitude processing was fully mediated by the performance in arithmetic tasks. We assume that a higher degree of familiarity with arithmetic in Chinese compared to German children leads to a higher speed of retrieving symbolic numerical magnitude knowledge.

## Introduction

According to the recently proposed “integrative theory of numerical development", numerical magnitude processing skills are at the core of numerical development and individual differences regarding these skills are assumed to be related to individual differences in arithmetic proficiency and math performance (Siegler and Lortie-Forgues, 2014). Numerical magnitude processing skills are typically assessed using magnitude comparison tasks. While non-symbolic numerical magnitude comparison tasks usually involve the comparison of two dot arrays, symbolic numerical magnitude comparison tasks involve the comparison of two Arabic digits. In either case, task difficulty is manipulated by varying the numerical distance between the stimuli to be compared. Task performance typically decreases in line with a decrease in numerical distance (e.g., Moyer and Landauer, 1967; Van Oeffelen and Vos, 1982).

Recent meta-analyses revealed a significant association between non-symbolic numerical magnitude processing skills and math performance (Chen and Li, 2014; Fazio et al., 2014) as well as between symbolic numerical magnitude processing skills and math performance (Schneider et al., 2015). It could be demonstrated that the association between non-symbolic numerical magnitude processing skills and math performance cannot entirely be attributed to general non-numerical cognitive abilities (e.g., Chen and Li, 2014). Moreover, based on the findings from longitudinal studies, Chen and Li (2014) report that while non-symbolic numerical magnitude processing skills prospectively predict later math performance, they can also be retrospectively predicted by earlier math performance. On the one hand, these findings are in line with the notion that non-symbolic numerical magnitude processing skills are fundamental to the development of mathematical skills, on the other hand they suggest that mathematical skills shape non-symbolic numerical magnitude processing skills. Schneider et al. (2015) compared the strength of the association between non-symbolic numerical magnitude processing skills and math performance with the strength of the association between symbolic numerical magnitude processing skills and math performance. The effect size was significantly higher for symbolic than for non-symbolic numerical magnitude processing skills. Longitudinal studies indicate that symbolic numerical magnitude processing skills are predictively related to mathematical skills (e.g., De Smedt et al., 2009; Vanbinst et al., 2015). This association cannot be explained by individual differences in children’s preschool mathematical abilities, intellectual abilities, processing speed, and verbal as well as visual-spatial short-term memory skills (Vanbinst et al., 2015). To our knowledge, however, the question of whether symbolic numerical magnitude processing skills may also be shaped by mathematical skills has not yet been examined. The association between symbolic numerical magnitude processing skills and more complex mathematical skills such as mental arithmetic is most consistently found for overall average reaction time in symbolic numerical magnitude comparison tasks, suggesting that children’s familiarity and fluency in manipulating symbolic numbers serves as the crucial link (Lyons et al., 2015). In addition, arithmetic problem solving is supposed to involve the retrieval of numerical magnitude knowledge (e.g., Siegler and Lortie-Forgues, 2014; Schneider et al., 2015), and thus a higher familiarity and fluency with arithmetic can be assumed to induce a higher familiarity and fluency in symbolic numerical magnitude processing.

Cross-national assessments of mathematical achievement have repeatedly demonstrated that Chinese children outperform their non-Chinese peers at various ages (e.g., Wang and Lin, 2009, 2013; Mullis et al., 2012; OECD, 2013). Hence, if a higher familiarity and fluency with arithmetic is reflected in a higher familiarity and fluency in symbolic numerical magnitude processing, a superior Chinese performance should not only exist for arithmetic skills but also for symbolic numerical magnitude processing skills. Recently, Rodic et al. (2014) compared 5 to 7-year old children from China, Kyrgyzstan, Russia, and the UK with regard to simple arithmetic tasks and different precursor skills assumed to be related to the development of arithmetic skills (i.e., non-symbolic numerical magnitude comparison, dot enumeration, number naming, and symbolic numerical magnitude comparison). In line with previous findings, Chinese children significantly outperformed all other groups in the arithmetic tasks. The superior arithmetic performance of Chinese children was, however, not (exactly) mirrored in the precursor skills. Russian children, for example, did not perform significantly worse than Chinese children in any of these measures. Nevertheless, only the understanding of symbolic number explained variation in arithmetic performance in all samples and was therefore regarded as the most important predictor of individual differences in early arithmetic by the authors (see Rodic et al., 2014). Conversely, a potential influence of arithmetic skills on the understanding of symbolic number was not addressed by Rodic et al. (2014). Indeed, the influence of symbolic number processing skills on arithmetic skills might be higher than the opposite direction of influence in the relevant age group because the children were only beginning to develop arithmetic skills.

To further explore the association between arithmetic skills and symbolic number magnitude processing skills, we tested Chinese and German third graders. In their 3rd year of elementary school, children typically possess basic arithmetic skills. If better arithmetic skills are reflected in symbolic numerical magnitude processing skills, a superior Chinese performance should not only exist for arithmetic skills but also for symbolic numerical magnitude processing skills. Moreover, if arithmetic skills shape symbolic number magnitude processing skills, a performance difference between Chinese and German children in symbolic numerical magnitude processing should be mediated by arithmetic skills. As a performance difference between Chinese and German children in the arithmetic tasks as well as in the symbolic numerical magnitude comparison task might be due to the fact that Chinese number words can be verbalized more quickly than German number words (e.g., Lüer et al., 1998), we also measured children’s performance in a task assessing speed of number pronunciation and included it as a control measure.

## Materials and Methods

### Participants

The German sample consisted of 33 third graders (18 female, mean age 9.1, range 8–10 years) recruited from a public primary school in Mühlheim am Main (Germany). The Chinese sample was the one described by Lonnemann et al. (2011): Participants were 33 (18 female) Chinese third graders (mean age 9.3, range 8–10 years) recruited from a public primary school in Shanghai (China). One Chinese child was excluded from further analysis because of exhibiting extreme scores in the addition task as well as in the symbolic numerical comparison task (addition *z*-score: -3.02, RT symbolic numerical magnitude comparison *z*-score: -1.75). Written and informed consent was obtained from the parents of all participating children.

### Tasks

All children started with the symbolic numerical magnitude comparison task, then proceeded to the arithmetic tasks, and finally worked on the task assessing speed of number pronunciation. All tasks were carried out individually.

### Symbolic Numerical Magnitude Comparison

In the symbolic numerical magnitude comparison task, two single-digit Arabic numbers were presented on a screen. The two stimuli were arranged in a horizontal fashion. Children had to indicate the side with the larger numerical magnitude by using the left index finger when it was larger on the left hand side and by using the right index finger when it was larger on the right hand side. Responses were given by pressing the ‘S’ and ‘L’ keys on a notebook keyboard. Comparison pairs varied along four numerical distances (see **Table 1**). Each of the 12 comparison pairs was presented eight times, four times with the larger number on the left hand side and four times with the larger number on the right hand side. Reaction times (RT) and error rate (ER) were recorded and the instruction stressed both speed and accuracy. The trials were pseudo-randomized so that there were no consecutive identical comparison pairs and numerical distance was not identical on more than three consecutive trials. The experiment was preceded by six warm-up trials to familiarize participants with the task (data not recorded), and presented on a notebook with Presentation^®^ software (Neurobehavioral Systems, Inc.). Black-colored Arabic digits were presented in Times 60-point font on a 17^′′^ color screen against a white background. A target stimulus was presented until the response was given but only up to a maximum duration of 4000 milliseconds (ms), and was followed by a black screen for 700 ms. If no response was given, a trial was classified as erroneous. Correct responses were used for computing mean RT. Response times below 200 ms were excluded from further analysis as well as responses outside an interval of ±3 standard deviations around the individual mean. Trimming resulted in 1.5% of response exclusions for Chinese participants and in 1.3% of response exclusions for German participants. A reciprocal transformation (dividing 1 by each score) was carried out on mean RT to yield more normally distributed data (the Shapiro–Wilk test revealed that the distribution was not significantly different from a normal distribution after transformation, for Chinese participants *p* = 0.19; for German participants *p* = 0.25). To estimate the reliability of the symbolic numerical magnitude comparison task, the Pearson correlation coefficient between reciprocal RT in odd and even trials was computed separately for Chinese and German participants (Chinese participants: *r* = 0.97; German participants: *r* = 0.97).

**Table 1:** Comparison pairs for the different numerical distances.

Distance			
**1**	**2**	**3**	**4**
1–2	1–3	2–5	1–5
2–3	2–4	3–6	2–6
4–5	3–5		
5–6	4–6		

### Arithmetic

The arithmetic tasks consisted of nine blocks of ten problems each (see Lonnemann et al., 2008, 2011); five blocks were addition problems and four blocks were subtraction problems. The addition problems were divided into two blocks in which a single-digit number had to be added to a two-digit number (e.g., 82 + 5), with only one of these blocks requiring carrying (e.g., 43 + 9). Moreover, three blocks contained addition problems in which two two-digit numbers had to be added (e.g., 24 + 65). In only one of these latter blocks, one of the addends was a decade number (e.g., 68 + 30). Among the remaining two blocks without decade numbers, again, only one block required carrying (e.g., 13 + 88). The subtraction problems were structured in a similar way: there were two blocks in which a single-digit number had to be subtracted from a two-digit number (e.g., 25 – 3) and two blocks which required subtraction of a two-digit number from another (e.g., 76 – 23). In both cases, only one block required borrowing (e.g., 54 – 7 or 82 – 45). Children were asked to write down as many solutions as possible in 30 s per block. Total scores ranging from 0 to 90 were used to estimate arithmetic skills. To estimate the reliability of the arithmetic tasks, *Cronbach’s alpha* was computed separately for Chinese and German participants (Chinese participants: *Cronbach’s α* = 0.69; German participants: *Cronbach’s α* = 0.91).

### Speed of Number Pronunciation

Children received two sheets of paper, each listing 60 Arabic digits. Stimuli were arranged in six rows of ten items and presented in Times New Roman 48-point font. Children were instructed to correctly name the items as quickly as possible and to proceed from left to right, starting at the top row and continuing to the bottom row. The first sheet contained the numbers 1–3 and the second one the numbers 4–6 with no consecutive identical stimuli. Response time was measured using a stopwatch from a start signal until the child named the last stimulus. The mean response time of both sheets was used to estimate speed of number pronunciation. To yield more normally distributed data, a reciprocal transformation (dividing 1 by each score) was carried out on mean response time (the Shapiro–Wilk test revealed that the distribution was not significantly different from a normal distribution after transformation, for Chinese participants *p* = 0.26; for German participants *p* = 0.28). To estimate the reliability of the speed of number pronunciation tasks, the Pearson correlation coefficient between the response times of both sheets was computed separately for Chinese and German participants (Chinese participants: *r* = 0.86; German participants: *r* = 0.84).

### Analyses

By using two-sample *t*-tests, Chinese and German children were compared with regard to reciprocal RT in the symbolic numerical magnitude comparison task, arithmetic skills, and reciprocal speed of number pronunciation. The Kolmogorov–Smirnov-*Z* test was used to compare age and ER in the symbolic numerical magnitude comparison task because the assumption of normality was violated for these variables.

To assess effects of the distance between the two to-be-compared Arabic digits in the symbolic numerical magnitude comparison task, we looked for linear trends based on reciprocal RT separately for Chinese and German participants. ER was low in the symbolic numerical magnitude comparison task and it did not significantly differ between groups (see **Table 2**) so it was not further analyzed.

**Table 2:** Comparison of Chinese and German children (paired-sample *t*-tests/Kolmogorov–Smirnov-*Z* tests) with respect to age (in month), reaction times (in ms) and errors (in %) in the symbolic numerical magnitude comparison task (RT comparison, ER comparison), arithmetic skills, and speed of number pronunciation (in seconds).

	Chinese children			German children			*p* (two-sided)
	*M*	*SD*	*SE*	*M*	*SD*	*SE*	
Age	111	4.13	0.73	109	5.94	1.03	*p* = 0.22
RT comparison^∗^	656	115.12	20.35	812	161.71	28.15	*p* < 0.001
ER comparison	2.86	2.05	0.36	2.21	2.24	0.39	*p* = 0.22
Arithmetic	78	7.70	1.36	37	13.64	2.37	*p* < 0.001
Speed of number pronunciation^∗^	28	6.68	1.18	37	7.68	1.34	*p* < 0.001

*n = 65 (32 Chinese and 33 German children); ^∗^p-value based on analysis of reciprocal reaction/response times.*

In order to test whether a possible performance difference between Chinese and German children in the symbolic numerical magnitude comparison task was mediated by arithmetic skills, we used mediation analyses. On the one hand, mediation analysis allows to investigate direct associations, used in this study to examine the relation between the factor group (Chinese vs. German) and individual performance in the symbolic numerical magnitude comparison task, while holding constant the performance in the arithmetic tasks. On the other hand, mediation analysis provides estimates of the statistical significance of indirect associations, used in this study to evaluate whether arithmetic skills mediate the association between the factor group and symbolic numerical magnitude processing skills. A second mediation model was tested to check the opposite direction of influence, i.e., to examine whether a possible performance difference between Chinese and German participants in the arithmetic tasks was mediated by the performance in the symbolic numerical magnitude comparison task. The mediation models were tested using the INDIRECT macro in SPSS (Preacher and Hayes, 2008). This macro uses the bootstrapping method with bias-corrected confidence estimates. Confidence intervals (95%) for the indirect associations were obtained using 5000 bootstrap samples. If a confidence interval does not include zero, the indirect effect is deemed statistically different from zero representing evidence for a mediating effect (Hayes and Preacher, 2014). Reciprocal speed of number pronunciation was used as control variable in the mediation analyses.

Moreover, Pearson correlation coefficients (before and after correction for attenuation) were employed to verify associations between arithmetic skills and reciprocal RT in the symbolic numerical magnitude comparison task as well as between arithmetic skills and reciprocal speed of number pronunciation, separately in both groups. The respective correlation coefficients of both groups were compared directly using the Fisher *r*-to-*z* transformation. All effects were tested using a significance level of α = 0.05.

## Results

While Chinese and German children did not significantly differ with regard to age (*Z* = 1.05, *p* = 0.22) and ER in the symbolic numerical magnitude comparison task (*Z* = 1.05, *p* = 0.22), Chinese children showed faster responses in the symbolic numerical magnitude comparison task [*t*(63) = 4.85, *p* < 0.001, *d* = 1.11], better arithmetic skills [*t*(51) = 14.77, *p* < 0.001, *d* = 3.70], and a higher speed of number pronunciation [*t*(48) = 5.53, *p* < 0.001, *d* = 1.25; see **Table 2**]. As Levene’s test indicated unequal variances for the performance in the arithmetic tasks (*F* = 7.20, *p* = 0.009) and for the speed of number pronunciation (*F* = 14.83, *p* < 0.001), degrees of freedom were adjusted.

**FIGURE 1:**
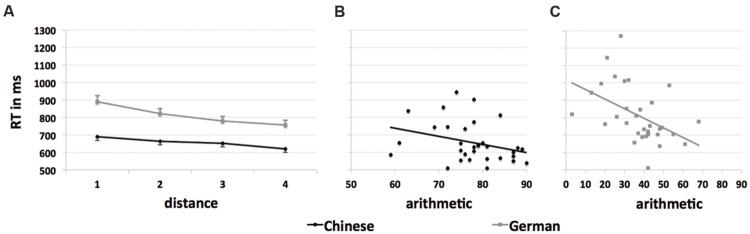
**Reaction times (RT in ms) for correct responses in the symbolic numerical magnitude comparison task and correlations with the performance in the arithmetic tasks. (A)** RT separately for Chinese and German children as a function of the factor distance (1, 2, 3, 4). **(B,C)** Correlations between the performance in the arithmetic tasks (raw score totals, theoretical range: 0–90) and mean RT in the symbolic numerical magnitude comparison task separately for Chinese and German children.

Reaction times in the symbolic numerical magnitude comparison task increased as the numerical distance between the two to-be-compared Arabic digits decreased: significant linear trends were found for Chinese [*F*(1,31) = 77.51, *p* < 0.001, η_p_^2^ = 0.71] and for German children [*F*(1,32) = 104.34, *p* < 0.001, η_p_^2^ = 0.77; see **Figure 1A**].

**FIGURE 2:**
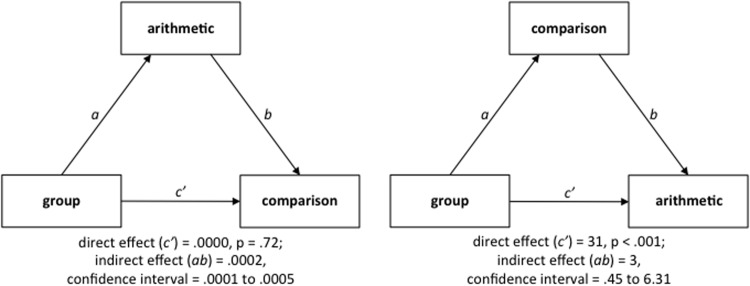
**Mediation models. (Left)** Model testing whether performance in the arithmetic tasks mediates the association between the factor group (Chinese vs. German) and reciprocal RT in the symbolic numerical magnitude comparison task. **(Right)** Model testing whether reciprocal RT in the symbolic numerical magnitude comparison task mediate the association between the factor group (Chinese vs. German) and performance in the arithmetic tasks.

The first mediation model revealed that the group difference in reciprocal RT in the symbolic numerical magnitude comparison task was no longer significant after controlling for arithmetic skills [direct effect = 0.0000, *t*(63) = 0.36, *p* = 0.72] and it was significantly mediated by the performance in the arithmetic tasks (indirect effect = 0.0002; confidence interval = 0.0001 to 0.0005; see **Figure 2**). Speed of number pronunciation had no significant partial effect on reciprocal RT in the symbolic numerical magnitude comparison task [*t*(63) = 1.03, *p* = 0.31]. The second mediation model showed that the group difference in arithmetic performance was still significant after controlling for reciprocal RT in the symbolic numerical magnitude comparison task [direct effect = 31, *t*(63) = 10.08, *p* < 0.001]. However, the group difference in arithmetic performance was significantly mediated by reciprocal RT in the symbolic numerical magnitude comparison task (indirect effect = 3; confidence interval = 0.45–6.31; see **Figure 2**). Moreover, speed of number pronunciation had a significant partial effect on arithmetic performance [*t*(63) = 3.54, *p* < 0.001].

For Chinese participants, reciprocal RT in the symbolic numerical magnitude comparison task was marginally correlated with performance in the arithmetic tasks (*r* = 0.32, *p* = 0.079 [two-sided], after correction for attenuation: *r* = 0.39, *p* = 0.027 [two-sided]), while for German participants a significant correlation was observed (*r* = 0.46, *p* = 0.007 [two-sided], after correction for attenuation: *r* = 0.49, *p* = 0.004 [two-sided], see **Figures 1B,C**). Reciprocal speed of number pronunciation and arithmetic skills were significantly correlated in both groups (Chinese children: *r* = 0.63, *p* < 0.001 [two-sided], after correction for attenuation: *r* = 0.82, *p* < 0.001 [two-sided]; German children: *r* = 0.49, *p* = 0.004 [two-sided], after correction for attenuation: *r* = 0.56, *p* < 0.001 [two-sided]). Comparison of the respective correlation coefficients of both groups did not reveal any significant differences (mean RT in the symbolic numerical comparison task and arithmetic skills: *r* = 0.32 vs. *r* = 0.46; *p* = 0.49 [two-sided]; speed of number pronunciation and arithmetic skills: *r* = 0.63 vs. *r* = 0.49; *p* = 0.43 [two-sided]).

## Discussion

We compared Chinese and German third graders regarding their performance in arithmetic tasks and in a symbolic numerical magnitude comparison task. Chinese children showed better performance in the arithmetic tasks, corresponding to previous findings (e.g., Wang and Lin, 2009, 2013; Mullis et al., 2012; OECD, 2013). This superior arithmetic performance of Chinese children was accompanied by a better performance of Chinese children in the symbolic numerical magnitude comparison task: Chinese children were overall faster in comparing two single-digit Arabic numbers with respect to their numerical magnitude without making more errors than German children. Thus, Chinese third graders not only showed a higher fluency in solving arithmetic tasks but were also able to compare Arabic digits at a faster pace than their German peers.

Mediation analysis revealed that the group difference in symbolic numerical magnitude processing was fully mediated by the performance in the arithmetic tasks. After controlling for arithmetic performance, the difference between Chinese and German children’s performance in the symbolic numerical magnitude comparison task was no longer significant. The difference between Chinese and German children in arithmetic was partially mediated by symbolic numerical magnitude processing skills. Indeed, the group difference in arithmetic performance was significantly mediated by the performance in the symbolic numerical magnitude comparison task but it was still significant after controlling for the performance in the symbolic numerical magnitude comparison task. Hence, while the group difference in arithmetic performance was only partially mediated by symbolic numerical magnitude processing skills, the group difference in symbolic numerical magnitude processing was fully mediated by the performance in the arithmetic tasks. The influence of arithmetic skills on symbolic numerical magnitude processing skills accordingly seems to be higher than the opposite direction of influence, at least in children who have already developed basic arithmetic skills. These findings might be seen as evidence for the notion that arithmetic skills shape symbolic numerical magnitude processing skills. Based on the assumptions that (a) children’s familiarity and fluency of manipulating symbolic numbers serves as the crucial link between symbolic numerical magnitude processing and arithmetic skills (Lyons et al., 2015), and (b) arithmetic problem solving involves the retrieval of numerical magnitude knowledge (e.g., Siegler and Lortie-Forgues, 2014; Schneider et al., 2015), we assume that a higher familiarity and fluency with arithmetic in Chinese compared to German children, most likely caused by a higher frequency of exposure to arithmetic (see e.g., Geary, 1996), leads to a higher speed of retrieving symbolic numerical magnitude knowledge. In addition, symbolic numerical magnitude processing skills seem to play a role in explaining the performance difference between Chinese and German third graders in arithmetic tasks. However, our findings suggest that other factors are also at play. Indeed, the speed of number pronunciation was found to be higher in Chinese children, most likely caused by the short length of Chinese number words, and it had a significant effect on arithmetic performance. This is in line with previous studies showing that the so-called “rapid automatized naming” (RAN) of Arabic digits or “number naming speed” is significantly correlated with arithmetic skills (e.g., Krajewski and Schneider, 2009). Moreover, besides educational practical factors like frequency of exposure to arithmetic, other possible explanations for the performance difference between Chinese and German children in arithmetic tasks might be found in the structure of number naming systems, cultural beliefs and values as well as parental involvement (e.g., Ng and Rao, 2010).

In accordance with previous findings, RT in the symbolic numerical magnitude comparison task correlated with arithmetic skills in German children (see e.g., Schneider et al., 2015). For Chinese children, by contrast, only a marginal correlation between RT in the symbolic numerical magnitude comparison task and arithmetic skills was found. A possible reason for these divergent findings might be that the between-subject variation in arithmetic performance was lower among Chinese participants (see **Table 2**; **Figures 1B,C**). However, comparing the respective correlation coefficients of both groups did not reveal any significant differences so that we should not assume any substantial between-group differences in the association between RT in the symbolic numerical magnitude comparison task and arithmetic performance.

It is important to note that the cross-sectional design of the current study does not offer means of assessing cause. Based on the different results of the two mediation models, we assume that a higher degree of familiarity and fluency with arithmetic in Chinese compared to German third graders causes a higher speed of retrieving symbolic numerical magnitude knowledge. To substantiate this notion, however, longitudinal studies are needed. The assessment of both the development of symbolic numerical magnitude processing skills and the development of arithmetic skills in Chinese and German children over time would lead to a better understanding of the interrelationship between these skills. Moreover, it would be possible to examine whether the direction of influence changes in the course of development and determine to what extent the developmental trajectories are culture-specific.

Another limitation of our study is that the two groups under study might have differed with respect to other factors that may account for the group differences in symbolic numerical magnitude processing and in arithmetic skills, but were not assessed in this study. For example, general cognitive abilities of Chinese and German children were not assessed. Instead of controlling for general cognitive abilities, we used a domain-specific control task, allowing us to rule out that our findings can be explained by between-group differences in the speed of number pronunciation. It can, however, not be ruled out that our findings are due to between-group differences in general intellectual abilities. Nonetheless, findings from previous studies do not support this notion but demonstrated that proficiency in comparing symbolic numbers is not related to children’s intellectual abilities (De Smedt et al., 2009; Vanbinst et al., 2012, 2015). Furthermore, findings from various studies suggest that the relationship between symbolic numerical magnitude processing and mathematics achievement cannot be explained by recourse to intelligence: first, the relationship was detected in typically developing children after controlling for intelligence (De Smedt et al., 2009; Vanbinst et al., 2012; Linsen et al., 2014). Second, compared to their typically developing peers, children with genetic syndromes that are associated with below-average intellectual and mathematical abilities are impaired in symbolic numerical magnitude processing after controlling for intelligence (22q11 deletion syndrome: De Smedt et al., 2007; Simon et al., 2008; Williams syndrome: O’Hearn and Landau, 2007). Third, children with developmental dyscalculia are impaired in symbolic numerical magnitude processing compared to their typically developing peers, after matching the groups on intelligence (Landerl et al., 2004; Ashkenazi et al., 2009; Mussolin et al., 2010; Desoete et al., 2012). Finally, it was demonstrated recently that children with mathematical difficulties and average intellectual abilities as well as children with mathematical difficulties and below-average intellectual abilities show similar impairments in symbolic numerical magnitude processing compared to controls. Furthermore, the difference on the symbolic numerical magnitude comparison task between children with mathematical difficulties and controls could not be explained by individual differences in working memory or general response speed (Brankaer et al., 2014). These findings, taken together, suggest that the present findings cannot be attributed to between-group differences in general cognitive abilities.

To conclude, results from our study revealed that differences in arithmetic performance between Chinese and German children are accompanied by differences in processing of symbolic numerical magnitude. Chinese third graders did not only show a higher fluency in solving arithmetic tasks but were also able to process symbolic numerical magnitude information at a faster pace than their German peers. The group difference in symbolic numerical magnitude processing was fully mediated by the performance in arithmetic tasks, suggesting that arithmetic skills shape symbolic numerical magnitude processing skills. We assume that a higher frequency of exposure to arithmetic leads to a higher degree of familiarity with arithmetic in Chinese compared to German children, in turn leading to a higher speed of retrieving symbolic numerical magnitude knowledge.

## Author Contributions

JL, JL, MH, and SL substantially contributed to the conception and design of the work, the acquisition, analysis, and interpretation of data for the work. JL, JL, MH, and SL substantially contributed to drafting the work and revising it critically for important intellectual content. JL, JL, MH, and SL substantially contributed to final approval of the version to be published. JL, JL, MH, and SL agreed to be accountable for all aspects of the work in ensuring that questions related to the accuracy or integrity of any part of the work are appropriately investigated and resolved.

## Conflict of Interest Statement

The authors declare that the research was conducted in the absence of any commercial or financial relationships that could be construed as a potential conflict of interest.
